# Experimental Infection of Chickens with H5N8 High Pathogenicity Avian Influenza Viruses Isolated in Japan in the Winter of 2020–2021

**DOI:** 10.3390/v15122293

**Published:** 2023-11-23

**Authors:** Saki Sakuma, Taichiro Tanikawa, Ryota Tsunekuni, Junki Mine, Asuka Kumagai, Kohtaro Miyazawa, Yoshihiro Takadate, Yuko Uchida

**Affiliations:** National Institute of Animal Health, National Agriculture and Food Research Organization, 3-1-5 Kannondai, Tsukuba 305-0856, Ibaraki, Japan; sakumas438@affrc.go.jp (S.S.); ttanikawa@affrc.go.jp (T.T.); tune@affrc.go.jp (R.T.); minejun84032@affrc.go.jp (J.M.); kumagaia412@affrc.go.jp (A.K.); miyazawak@affrc.go.jp (K.M.); takadatey851@affrc.go.jp (Y.T.)

**Keywords:** avian flu, high pathogenicity avian influenza virus, Japan, infectivity, virulence, transmissibility, chicken

## Abstract

During the winter of 2020–2021, numerous outbreaks of high pathogenicity avian influenza (HPAI) were caused by viruses of the subtype H5N8 in poultry over a wide region in Japan. The virus can be divided into five genotypes—E1, E2, E3, E5, and E7. The major genotype responsible for the outbreaks was E3, followed by E2. To investigate the cause of these outbreaks, we experimentally infected chickens with five representative strains of each genotype. We found that the 50% chicken infectious dose differed by up to 75 times among the five strains, and the titer of the E3 strains (10^2.75^ 50% egg infectious dose (EID_50_)) was the lowest, followed by that of the E2 strains (10^3.50^ EID_50_). In viral transmission experiments, in addition to the E3 and E2 strains, the E5 strain was transmitted to naïve chickens with high efficiency (>80%), whereas the other strains had low efficiencies (<20%). We observed a clear difference in the virological characteristics among the five strains isolated in the same season. The higher infectivity of the E3 and E2 viruses in chickens may have caused the large number of HPAI outbreaks in Japan during this season.

## 1. Introduction

Since the first detection of the H5N1 high pathogenicity avian influenza virus (HPAIV) A/goose/Guangdong/1/1996 (Gs/Gd) in China in 1996 [1], H5 HPAIVs of the Gs/Gd lineage have been found in several countries, excluding Oceania, for a quarter of a century, causing serious damage to poultry farming [2,3]. To date, the Gs/Gd lineage H5 HPAIVs have evolved into 10 genetically distinct hemagglutinin (HA) clades (0–9) and multiple subclades [4,5,6] and have reassorted with other avian influenza viruses [3]. Unlike other clades, clade 2.3.4.4, to which H5 HPAIVs belong, has acquired N1, N2, N3, N4, N5, N6, N8, and N9 neuraminidases (NA) via reassortment events [7,8], causing global outbreaks in poultry and wild birds since 2014 [3,9].

In the winter of 2020–2021, the Gs/Gd lineage H5 HPAIVs were detected in poultry and wild birds in Asia, Europe, and Africa, and most of the poultry outbreaks were caused by H5N8 HPAIVs belonging to clade 2.3.4.4b [10]. In the phylogenetic tree of HA genes, H5 HPAIVs of clade 2.3.4.4b isolated in this season belonged to two distinct clusters: one primarily comprising HPAIVs in Europe in early 2020 and HPAIVs in Asia in late 2020; the other primarily comprising HPAIVs in Europe in late 2020 [10]. Baek et al. [11] named the abovementioned HA clusters as G1 and G2 clusters, respectively, and classified Korean HPAIVs that were isolated in the winter of 2020–2021 into seven distinct genotypes by constellations of all gene segments, in which HA genes of the G1 cluster were divided into E1 and E3–E7 genotypes, and those of the G2 cluster were designated as the E2 genotype. In the winter of 2020–2021, the first poultry outbreak was caused by E1-genotype HPAIV in Japan [12]. During the winter of 2020–2021, 52 HPAI outbreaks in poultry farms and 58 HPAI cases from wild birds/environment were reported [13,14,15]. Phylogenetic analysis of Japanese strains associated with the 52 outbreaks in poultry farms and 16 of 58 detected cases from wild birds/the environment showed that they possessed HA genes of clade 2.3.4.4b and were divided into five distinct genotypes (E1, E2, E3, E5, and E7) [13,14,15]. The prevalence of HPAIVs in poultry was shown to be different among the genotypes, in which HPAIVs of the E3 and E2 genotypes caused 23 and 12 poultry outbreaks, respectively, over a large area; HPAIVs of the E1 genotype caused 11 poultry outbreaks in a very limited area, and HPAIVs of the E5 and E7 genotypes caused one and five poultry outbreaks, respectively [13]. In addition, two novel genotypes of HPAIVs, the E8 and E9 genotypes, have been isolated and reported recently from environmental water collected in Japan during the winter of 2020–2021 [16].

The infectivity and virulence of an E1 strain, A/chicken/Kagawa/11C/2020 (Kagawa11C), isolated from the first outbreak in chickens have been reported [12]. The infectivity and virulence of Japanese-origin H5N8 HPAIVs belonging to other genotypes are not yet clear. In the present study, we investigated those of HPAIVs of the other four genotypes that caused poultry outbreaks (E2, E3, E5, and E7) in chickens by assessing the virus dose required for infection, survivability, and virus shedding. Furthermore, we assessed the transmissibility of HPAIVs of the five genotypes in chickens. We aimed to investigate the association between HPAI poultry outbreaks in Japan in the winter of 2020–2021 and the virological characteristics of HPAIVs of the five genotypes that caused poultry outbreaks.

## 2. Materials and Methods

### 2.1. Viruses

One representative strain was selected from the outbreaks in poultry by four genotypes, E1, E2, E3, and E7, and from the case in wild birds by the E5 genotype for animal experiments (Table 1). A representative strain of the E1 genotype, A/chicken/Kagawa/11C/2020 (Kagawa11C), which has been previously described [12], was used in the transmissibility experiment. The representative strains of the other genotypes were as follows: A/duck/Chiba/C1T/2021 (ChibaC1T), E2 genotype; A/chicken/Fukuoka/T1/2020 (FukuokaT1), E3 genotype; A/eastern_buzzard/Toyama/160213T/2021 (Toyama16T), E5 genotype; and A/chicken/Tokushima/4T/2020 (Tokushima4T), E7 genotype. These Japanese strains were isolated as previously described [13].

### 2.2. Animal Experiments

White Leghorn chickens (L-M-6 strain) that had not been exposed to influenza A virus were obtained from Nisseiken Co., Ltd. (Tokyo, Japan). To ensure that they were serologically negative for influenza A virus, serum samples were collected from all birds before they were used in subsequent animal experiments, and serum antibodies against this virus were examined using the Influenza A Virus Antibody Test Kit (IDEXX Laboratories, Westbrook, ME, USA). All animal experiments were performed in Biosafety Level 3 facilities at the National Institute of Animal Health, Japan and were conducted in compliance with the institutional protocol, which was reviewed and approved by the Institutional Animal Care and Use Committee of the National Agriculture and Food Research Organization (approval number 20-078, 20 January 2021; approval number 20-084, 16 February 2021; approval number 21-001, 14 April 2021; approval number 21-026, 17 May 2021).

### 2.3. Pathogenicity

The pathogenicity of the ChibaC1T, FukuokaT1, Toyama16T, and Tokushima4T strains were determined based on the World Organization for Animal Health (WOAH) manual [17]. In total, 8 5- to 8-week-old chickens of each strain were intravenously inoculated with 200 µL of a 1/10 dilution of the infectious allantoic fluid of each virus (the inoculated virus doses after dilution are listed in Table S1), and the chickens were observed for 10 days post-inoculation (dpi).

### 2.4. Infectivity and Virulence

To evaluate the infectivity and virulence of ChibaC1T, FukuokaT1, Toyama16T, and Tokushima4T, 5 or 6 4-week-old chickens of each strain were intranasally inoculated with the viruses at 50% egg infectious dose (EID_50_)/100 µL of 10^2^, 10^4^, 10^5^, or 10^6^ and then observed for 14 dpi. In the ChibaC1T and FukuokaT1 groups, the values of the 50% chicken lethal dose (CLD_50_) were calculated between 10^2^ and 10^4^ EID_50_/100 µL based on results of the inoculation with above viral doses. To more accurately calculate the CLD_50_, another 5 4-week-old chickens were inoculated with ChibaC1T and FukuokaT1 at a dose of 10^3^ EID_50_/100 µL. To assess their viral shedding during the 14-day period, tracheal and cloacal swabs were collected at 1, 2, 3, 5, 7, 10, and 14 dpi (or at the time of death). The swabs were dipped in 2.0 mL of minimum essential medium containing 0.5% bovine serum albumin, 25 µg/mL amphotericin B, 1000 units/mL penicillin, 1000 µg/mL streptomycin, 0.01 M HEPES, and 8.8 mg/mL NaHCO_3_. After the swabs were removed from the medium, the inoculated medium was stored at −80 °C until viral titration. The viral titers were calculated as EID_50_ using the Reed and Muench method [18]. The detection limit was 0.2 log_10_ EID_50_/mL. To assess the history of infection, serum samples were collected from the surviving chickens at the end of the experiment (14 dpi). Serum antibodies against influenza A virus were examined as described above.

### 2.5. Transmissibility

To evaluate the transmissibility of Kagawa11C, ChibaC1T, FukuokaT1, Toyama16T, and Tokushima4T in chickens, 7 4-week-old chickens of each strain were used. One chicken was intranasally inoculated with the virus at a dose of 10^6^ EID_50_. After 18 h, six naïve chickens were cohabited with the inoculated chicken and observed for 20 days post-cohabitation. Tracheal and cloacal swabs were collected at the time of cohabitation (only from the inoculated chickens) and at 1, 2, 4, 6, 9, 13, 16, and 20 days post-cohabitation (or at the time of death). Virus titration and verification of the history of infection in surviving chickens at the end of the experiment (20 days post-cohabitation) were performed as described above.

### 2.6. Statistical Analysis

To compare total virus shedding, the area under the curve (AUC) connecting the virus titers at each sampling point was calculated as described previously [19]. The differences in viral shedding among the five strains were evaluated using the Kruskal–Wallis test and the Steel–Dwass test using Statcedl4 ver. 4. (The Publisher OMS Ltd., Saitama, Japan). The differences in the survival curves were evaluated using the log-rank test adjusted by the Holm method using EZR (Saitama Medical Center, Jichi Medical University, Saitama, Japan) [20]. A *p*-value of <0.05 was considered statistically significant.

## 3. Results

### 3.1. Virological Characteristics of Japanese H5N8 HPAIVs in Chickens

**Figure 1:**
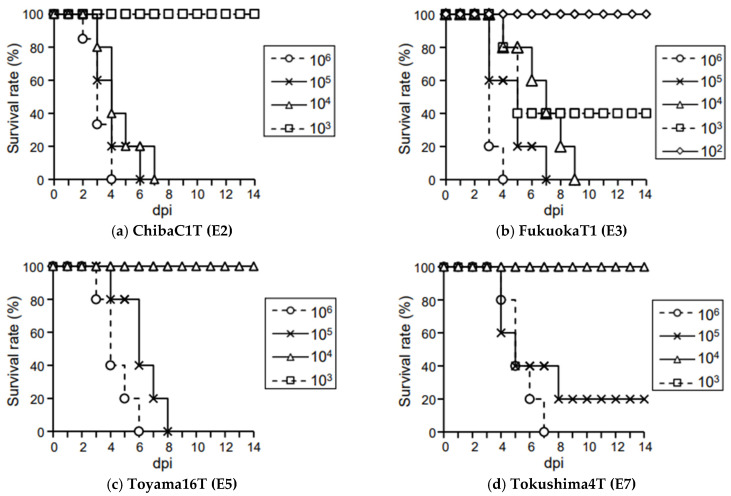
Survival rates of the chickens intranasally inoculated with (**a**) ChibaC1T, (**b**) FukuokaT1, (**c**) Toyama16T, and (**d**) Tokushima4T 14 days post-inoculation (dpi).

**Figure 2:**
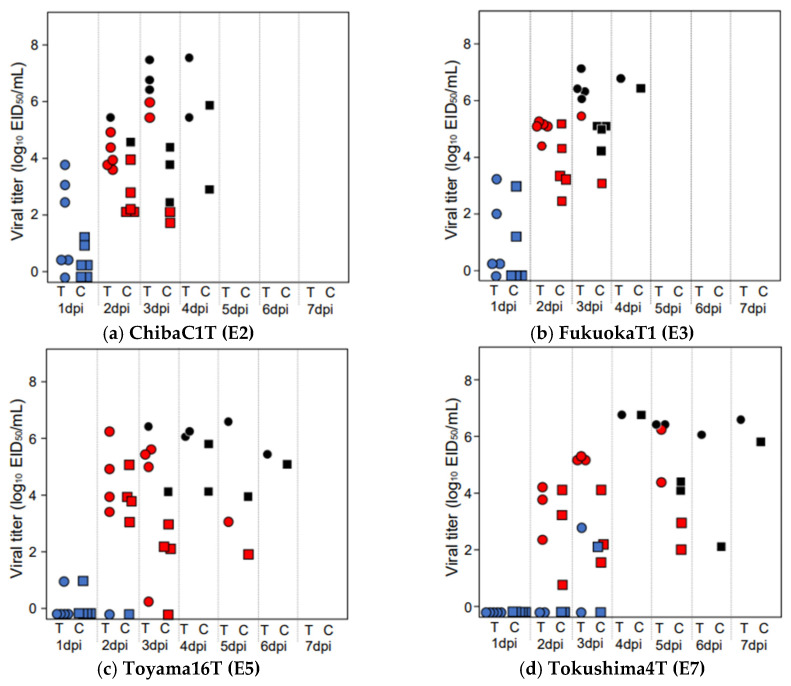
Viral titers in tracheal (T) or cloacal (C) swabs collected from the chickens intranasally inoculated with 10^6^ EID_50_ of (**a**) ChibaC1T, (**b**) FukuokaT1, (**c**) Toyama16T, and (**d**) Tokushima4T up to 7 days post-inoculation (dpi). Circles and squares represent the viral titers in tracheal and cloacal swabs, respectively. The colors of symbols represent the conditions of chickens: blue, live chickens without any clinical symptoms; red, live chickens with clinical signs; black, dead chickens.

In the present study, we investigated the infectivity and virulence of Japanese H5N8 HPAIVs classified into four genotypes: ChibaC1T (E2 genotype), FukuokaT1 (E3 genotype), Toyama16T (E5 genotype), and Tokushima4T (E7 genotype). When chickens were intravenously inoculated with each strain, all chickens died within 48 h post-inoculation (hpi) (Table S1), indicating that these strains were HPAIVs according to the criteria established by the WOAH. When five or six chickens were intranasally inoculated with several doses of each strain and observed for 14 dpi, their survival was as follows. In all groups, all chickens inoculated with the virus at a dose of 10^2^ EID_50_ survived the observation period (data not shown, except for FukuokaT1 in Figure 1b). In the ChibaC1T group, all chickens inoculated with viruses at doses of 10^6^, 10^5^, or 10^4^ EID_50_ died within 4, 6, or 7 dpi, respectively, whereas no chicken inoculated with 10^3^ EID_50_ died during the observation period (Figure 1a). In the FukuokaT1 group, all chickens inoculated with the viruses at doses of 10^6^, 10^5^, or 10^4^ EID_50_ died within 4, 7, or 9 dpi, respectively, and three of the five chickens inoculated with 10^3^ EID_50_ died within 5 dpi (Figure 1b). In the Toyama16T group, all chickens inoculated with viruses at doses of 10^6^ or 10^5^ EID_50_ died within 6 or 8 dpi, respectively, whereas no chickens inoculated with 10^4^ EID_50_ died during the observation period (Figure 1c). In the Tokushima4T group, all chickens inoculated with the virus at a dose of 10^6^ EID_50_ died within 7 dpi, and four of five chickens inoculated with 10^5^ EID_50_ died within 8 dpi (Figure 1d). No chickens inoculated with 10^4^ EID_50_ died during the observation period (Figure 1d). The 50% CLD_50_ values of ChibaC1T, FukuokaT1, Toyama16T, and Tokushima4T were 10^3.50^, 10^2.75^, 10^4.50^, and 10^4.63^ EID_50_, respectively (Table 2). The mean death times (MDTs) of the chickens inoculated with ChibaC1T, FukuokaT1, Toyama16T, and Tokushima4T at 10^6^ EID_50_ were 3.17, 3.20, 4.40, and 5.40 dpi, respectively (Table 2). Almost all exhibited various clinical signs, such as depression and cyanosis of the comb and wattles, from 2 dpi (ChibaC1T:6/6; FukuokaT1:5/5; Toyama16T:4/5; Tokushima4T; 3/5) (Figure 2). Among the five tested Japanese strains, including Kagawa11C [12], the CLD_50_ of FukuokaT1 was the lowest and that of Kagawa11C and Tokushima4T was the highest, with a difference of up to 75 times. The MDT in the ChibaC1T group was the shortest and that in the Kagawa11C group was the longest, with a difference of up to 2.43 days (Table 2). The survival curves of chickens inoculated with the viruses at a dose of 10^6^ EID_50_ were significantly different between ChibaC1T and Kagawa11C (log-rank test, *p* < 0.05), ChibaC1T and Tokushima4T (*p* < 0.05), FukuokaT1 and Kagawa11C (*p* < 0.05), and FukuokaT1 and Tokushima4T (*p* < 0.05). No chickens that survived during the observation period had any antibodies against influenza A virus and virus shedding (data not shown), indicating that the values of CLD_50_ corresponded to that of the chicken infectious dose (CID_50_) (Table 2).

### 3.2. Virus Shedding in Chickens Infected with HPAIVs at a Dose of 10^6^ EID_50_

To evaluate the dissemination of the virus from the infected chickens, we compared viral shedding from the trachea and cloaca in chickens infected with each virus at a dose of 10^6^ EID_50_ (Figure 2 and Table 2). When comparing viral shedding between the five groups, including the results of Kagawa11C [12], the shedding periods from the trachea and cloaca in the ChibaC1T, FukuokaT1, and Toyama16T groups (3 days) were shorter than those in the Tokushima4T (4 days) and Kagawa11C (5 days) groups (Table 2). In all groups, the viral titers in the trachea were higher than those in the cloaca (Table 2). In all groups, the maximum viral titer in the trachea during the observation period was detected in swabs collected on the day of death or the day before death. The titer in the ChibaC1T group (6.70 log_10_ EID_50_/mL) was the highest and followed by that in the FukuokaT1 and Tokushima4T (6.53 log_10_ EID_50_/mL), Toyama16T (6.32 log_10_ EID_50_/mL), and Kagawa11C (5.32 log_10_ EID_50_/mL) groups (Table 2). Regarding the AUC of the entire observation period used to compare total viral shedding, that of the trachea in the Tokushima4T group (3,901,427) was the highest, followed by that in the ChibaC1T (2,743,748), FukuokaT1 (1,916,183), Toyama16T (1,269,541), and Kagawa11C (163,355) groups (Table 2). Regarding the AUC from 1 to 3 dpi used to compare the total viral shedding in the early stages of infection, tracheal shedding in the FukuokaT1 group (1,359,707) was the highest, followed by that in the ChibaC1T (1,203,339), Toyama16T (261,854), Tokushima4T (112,761), and Kagawa11C (10,012) groups (Table 2). However, in the maximum viral titers and AUCs, there were no significant differences between the five tested Japanese strains.

### 3.3. Transmissibility of Japanese H5N8 Strains in Chickens

To evaluate the transmissibility of Japanese strains in chickens, we performed viral transmission experiments. In all strain groups, all virus-inoculated chickens died with viral shedding within 5 days of cohabitation (Figures S1 and S2). Almost all cohabiting chickens died in the ChibaC1T (6/6 chickens died between 4 and 8 days post-cohabitation), FukuokaT1 (5/6 chickens died between 5 and 8 days post-cohabitation), and Toyama16T (6/6 chickens died between 5 and 9 days post-cohabitation) groups, whereas no or almost no cohabiting chickens died in the Kagawa11C (1/6 chickens died 8 days post-cohabitation) and Tokushima4T (0/6 chickens) groups (Figure S1 and Table 3). The surviving cohabiting chickens during the observation period neither had any antibodies against the influenza A virus (data not shown) nor any viral shedding (Figure S2).

Regarding viral shedding in the infected cohabiting chickens, one of the six chickens in the ChibaC1T group started to shed viruses 2 days post-cohabitation (Figure S2b), and some virus-infected cohabiting chickens in the Kagawa11C, FukuokaT1, and Toyama16T groups started to shed viruses 4 days post-cohabitation (Figure S2a,c,d). In all groups, the viral titers in the trachea were higher than those in the cloaca (Table 3). When comparing viral shedding from virus-infected cohabiting chickens in the ChibaC1T, FukuokaT1, and Toyama16T groups, the shedding periods in the trachea of the ChibaC1T and FukuokaT1 groups (3 d) were longer than those in the Toyama16T group (2 d) (Table 3). In the maximum viral titer during the observation period in the trachea, that in the FukuokaT1 group (7.32 log_10_ EID_50_/mL) was the highest, followed by those in the ChibaC1T (6.76 log_10_ EID_50_/mL) and Toyama16T (6.11 log_10_ EID_50_/mL) groups (Table 3). In the AUC of the entire observation period, the value in the trachea in the FukuokaT1 group (21,085,137) was the highest, followed by that in the ChibaC1T (7,407,674) and Toyama16T (1,570,800) groups (Table 3). The differences in the maximum viral titers and AUCs in tracheal swabs between the FukuokaT1 and Toyama16T groups were significant (Steel–Dwass test, *p* < 0.05). In addition, the maximum viral titers for cloacal swabs between the Toyama16T and ChibaC1T groups were significantly different (Steel–Dwass test, *p* < 0.05).

## 4. Discussion

In the present study and a previous study [12], we investigated the infectivity, virulence, and transmissibility of five Japanese H5N8 HPAIVs isolated in the winter of 2020–2021 by chicken infection. Their infectivity was determined based on the CID_50_ value and virulence based on the MDTs. The infectivity and virulence of FukuokaT1 and ChibaC1T were higher than those of the other strains. The CID_50_ value of FukuokaT1 was the lowest among the five groups (10^2.75^ EID_50_), followed by that of ChibaC1T (10^3.50^ EID_50_) (Table 2). The values were notably lower in these two strains than in the other strains (Toyama16T: 10^4.50^ EID_50_; Kagawa11C and Tokushima4T: 10^4.63^ EID_50_) (Table 2). In addition, the MDTs of the chickens inoculated with each virus at a dose of 10^6^ EID_50_ in the FukuokaT1 and ChibaC1T groups (3.20 and 3.17 dpi) were shorter than those in the Toyama16T group (4.40 dpi) and the other two groups (Tokushima4T: 5.40 dpi; Kagawa11C: 5.60 dpi) (Table 2). The long survival periods of chickens infected with Kagawa11C and Tokusima4T prolonged viral shedding (Table 2). However, the AUCs of the entire observation period for the trachea in the FukuokaT1 and ChibaC1T groups were relatively high (Table 2). The tracheal AUC of the early stages of infection in the FukuokaT1 and ChibaC1T groups were much higher than those in the other three groups (Table 2), indicating that they can grow more efficiently in chickens. Viral transmission experiments revealed that Toyama16T, FukuokaT1, and ChibaC1T showed higher transmissibility in chickens than the other two strains. In virus-infected chickens, these viruses could be transmitted to other naïve chickens with more than 80% efficiency and efficiently propagated in chickens, whereas Kagawa11C and Tokushima4T could not be efficiently transmitted (Table 3). The high transmissibility of FukuokaT1 and ChibaC1T in chickens may be attributed to their higher infectivity. However, the high transmissibility of the Toyama16T strain was attributed to another reason, i.e., its low infectivity (Table 2). Factors not clarified in this study, such as the persistence of the virus in the environment, may have influenced its transmissibility. In the transmissibility experiments, naïve cohabiting chickens were infected with the virus via direct or indirect contact with virus-infected chickens. The titers of viral shedding from the cloaca in the Toyama16T group were the second highest among the five genotypes (Table 2). In the transmissibility experiments, a large amount of virus may have been shed into the environment from Toyama16T-infected chickens via fecal matter. Drinking water is regarded as a route of contact for the virus in the environment, and the tenacity of the virus in water varies according to the strains [21,22]. Further studies are required to identify the factors that increase the transmissibility of the virus.

In the field, the most outbreaks in poultry farms were caused by HPAIVs of the E3 genotype (23 of 52 cases) and followed by viruses of the E2 genotype (12 of 52 cases), which occurred over a wide area from east to south Japan (Table 1 and Figure S3b,c) [13]. Outbreaks of the E1 genotype in poultry were restricted to a small area of west Japan (Table 1 and Figure S3a) [13]. The number of poultry outbreaks for the E5 and E7 genotypes appeared to be relatively limited compared to those for other genotypes (Table 1 and Figure S3d,e) [13]. Associating the prevalence of HPAIVs in poultry with our findings in the present and previous studies [12], the high infectivity of viruses in chickens was suggested as a possible factor that increased the number and/or area of outbreaks in poultry. The E2 (ChibaC1T) and E3 (FukuokaT1) genotypes, which have higher infectivity, virulence, and transmissibility in chickens than the other three genotypes, have caused a large number of outbreaks in poultry farms over a wide area in Japan. In particular, the E3 genotype (FukuokaT1), which was the most infectious, caused approximately half of the outbreaks during the winter season. In contrast, poultry outbreaks caused by the three low-infectious genotypes, E1 (Kagawa11C), E5 (Toyama16T), and E7 (Tokushima4T), were limited in number or area, regardless of other virological characteristics. Given their lower infectivity, even if the viruses entered the farm from outside, it may have been difficult for the viruses in the farm environment to infect chickens, resulting in the restricted occurrence of outbreaks in poultry farms.

Migratory wild birds have been considered to play an important role in the global spread of HPAIVs [23,24,25,26]. HPAIV-infected migratory wild birds contaminate the environment by shedding viruses over breeding and wintering grounds during their migration, probably resulting in viral infections in other wild birds, including resident birds, via direct or indirect contact. Some studies with experimental infection with wild birds have reported that the susceptibility to HPAIVs was different among bird species [19,25,27,28,29,30,31,32]. In addition, during the autumn–spring seasons in Japan, several species of migratory wild birds have been reported [33,34]. The susceptibility to HPAIVs of wild bird species that migrated to Japan, their migratory routes and timing, and their habitats may also have influenced the differences in the number and period of poultry outbreaks among these five genotypes. Further studies on the susceptibility to HPAIVs in wild bird species and avian influenza virus surveillance in wild birds are required to predict and prepare for future HPAI outbreaks in Japan.

Here, we demonstrated that among the five genotypes of H5N8 HPAIVs detected in Japanese poultry farms in the winter of 2020–2021, E3 and E2 viruses had relatively higher infectivity, virulence, and transmissibility in chickens than the other genotypes. Especially, the E3 virus can easily infect chickens and efficiently propagate in chickens compared to almost all other viruses responsible for the past outbreaks [12]. This result suggests that the large number of HPAI outbreaks over a wide area of Japan during the winter of 2020–2021 may be, at least in part, associated with the infectivity of the E3 and E2 viruses in chickens. To prepare for future HPAI outbreaks, it is important to understand the virological characteristics of the viruses responsible for past outbreaks. Further elucidation of the genetic characteristics associated with these findings is required.

## Figures and Tables

**Table 1:** H5N8 HPAIV strains used in animal experiments and HPAI outbreak situation in Japan in 2020–2021.

Virus Strain	Genotype	Outbreaks in Japan in 2020–2021 ^a^					
		Bird Type	Number of Occurrences	Number of Prefectures Where Outbreaks Occurred	Date of Occurrence		
					First	Last	Period (Days)
Kagawa11C	E1	Poultry	11	2	2020.11.04	2020.12.01	27
		Wild birds/ environment	4	4	2020.10.24	2020.12.23	60
ChibaC1T	E2	Poultry	12	3	2021.01.20	2021.02.14	25
		Wild birds/ environment	4	3	2021.01.16	2021.02.14	29
FukuokaT1	E3	Poultry	23	13	2020.11.24	2021.03.13	109
		Wild birds/ environment	2	2	2020.12.22	2021.02.24	64
Toyama16T	E5	Poultry	1	1	2021.02.24	-	-
		Wild birds/ environment	1	1	2021.02.10	-	-
Tokushima4T	E7	Poultry	5	4	2020.12.18	2021.02.01	45
		Wild birds/ environment	5	2	2021.02.08	2021.03.03	23

^a^ The information of HPAI cases in poultry and wild birds/environment is quoted from the Ministry of the Agriculture, Forestry and Fisheries of Japan website “https://www.maff.go.jp/j/syouan/douei/tori/ (accessed on 8 August 2023)” and the Ministry of the Environment of Japan website “https://www.env.go.jp/nature/dobutsu/bird_flu/index.html (accessed on 8 August 2023)”, respectively. The genotype classification of all 52 cases in poultry and 16 of 58 cases in wild birds/environment is quoted from a previous study [13,14,15].

**Table 2:** Virological characteristics of Japanese H5N8 HPAIV strains in chickens.

Virus Strain	Genotype	CLD_50_^a^ (log_10_EID_50_)	Chickens Inoculated with HPAIVs at a Dose of 10^6^ EID_50_					
			MDT (dpi) ^b^	Viral Shedding of Chickens Inoculated with HPAIVs at a Dose of 10^6^ EID_50_				
				Swab	Shedding Period (days) ^c^	Maximum Viral Titer (log_10_ EID_50_/mL) ^d^	Area under the Viral Shedding Curve (AUC) ^d^	
							AUC of the Entire Observation Period	AUC of the Early Stages of Infection (1–3 dpi)
Kagawa11C ^e^	E1	4.63	5.60 (4–6)	Trachea	5 (4–5)	5.32 (4.32–6.01)	163,355 (60,078–681,723)	10,012 (2099–94,938)
				Cloaca	5 (3–5)	4.32 (3.87–6.02)	41,285 (7419–574,373)	1638 (340–7339)
ChibaC1T	E2	3.50	3.17 (2–4)	Trachea	3 (1–4)	6.70 (5.53–7.70)	2,743,748 (340,644–25,348,652)	1,203,339 (178,330–17,073,701)
				Cloaca	3 (2–4)	4.16 (2.53–6.02)	15,162 (346–520,507)	2378 (189–49,999)
FukuokaT1	E3	2.75	3.20 (3–4)	Trachea	3 (2–4)	6.53 (6.20–7.20)	1,916,183 (948,681–8,146,128)	1,359,707 (204,386–8,146,128)
				Cloaca	3 (2–3)	5.20 (4.32–6.53)	103,086 (13,371–1,705,148)	82,347 (1129–270,130)
Toyama16T	E5	4.50	4.40 (3–6)	Trachea	3 (3–4)	6.32 (5.53–6.70)	1,269,541 (172,692–3,811,719)	261,854 (0–3,811,719)
				Cloaca	3 (2–4)	5.20 (4.02–5.87)	79,210 (12,695–369,713)	7416 (0–158,217)
Tokushima4T	E7	4.63	5.40 (4–7)	Trachea	4 (3–4)	6.53 (6.32–6.87)	3,901,427 (1,921,413–5,068,124)	112,761 (0–126,507)
				Cloaca	4 (3–4)	4.53 (2.20–6.87)	36,484 (489–3,701,117)	78 (0–23,715)

^a^ CLD_50_, 50% chicken lethal dose. CLD_50_ corresponded to that of chicken infectious dose (CID_50_). ^b^ MDT, mean death time; dpi, days post-inoculation; numbers in parentheses, range of time to death. ^c^ The values are given as the median. Numbers in parentheses, range of all chickens. ^d^ The values are given as the median. Numbers in parentheses, range of all chickens. ^e^ The results for Kagawa11C are quoted from a previous study [12].

**Table 3:** Transmission rate of Japanese H5N8 HPAIVs in chickens and viral shedding in the virus-infected chickens in viral transmission experiments.

Virus Strain	Genotype	Transmission Rate (%) ^a^	Viral Shedding in the Virus-Infected Cohabiting Chickens ^b^			
			Swab	Shedding Period (Days)	Maximum Viral Titer (log_10_ EID_50_/mL)	Area under the Viral Shedding Curve (AUC)
Kagawa11C	E1	16.7 (1/6)	Trachea	5	6.32	531,852
			Cloaca	5	5.01	361
ChibaC1T	E2	100 (6/6)	Trachea	3 (1–3)	6.76 (5.45–7.87)	7,407,674 (281,323–73,394,176)
			Cloaca	2.5 (1–3)	3.77 (2.87–4.70)	9391 (1151–49,998)
FukuokaT1	E3	83.3 (5/6)	Trachea	3 (2–5)	7.32 (7.02–7.87)	21,085,137 (6,979,946–50,148,240)
			Cloaca	3 (2–4)	5.02 (3.53–5.70)	83,638 (3433–568,124)
Toyama16T	E5	100 (6/6)	Trachea	2 (1–3)	6.11 (5.32–6.32)	1,570,800 (105,424–3,529,670)
			Cloaca	1 (1–3)	5.09 (4.20–5.87)	145,522 (10,542–1,100,847)
Tokushima4T	E7	0 (0/6)	Trachea	-	-	-
			Cloaca	-	-	-

^a^ Numbers in parentheses, number of positive chickens/total chickens. ^b^ The data were calculated using the values of positive chickens. The values are given as the median. The numbers in parentheses are the range of positive chickens.

## Data Availability

The data that support this study are available from the corresponding author upon reasonable request.

## Supplementary Materials

The following supporting information can be downloaded at: https://www.mdpi.com/article/10.3390/v15122293/s1, Figure S1: Survival rates of chickens in the transmission experiment; Figure S2: Viral shedding of chickens in the transmission experiment; Figure S3: Locations and number of HPAI outbreaks in poultry farms in Japan during the winter of 2020–2021. Table S1 Survival rates of chickens intravenously inoculated with ChibaC1T, FukuokaT1, Toyama16T, or Tokushima4T.
