# Supplementary material for: Experimental Infection of Chickens with H5N8 High Pathogenicity Avian Influenza Viruses Isolated in Japan in the Winter of 2020–2021
Source: Viruses. 2023 Nov 23;15(12):2293. doi: 10.3390/v15122293 (PMC10748181; doi:10.3390/v15122293)
Supplement: Supplementary file 1 [file viruses-15-02293-s001.zip › viruses-2745102-supplementary.pdf]

Table S1. Survival rates of chickens intravenously inoculated with ChibaC1T, FukuokaT1, Toyama16T, or Tokushima4T

Virus strain	Genotype	Inoculated viral dose	Survival rate (%)[a]				
		(log_{10} EID_{50}/200 μL)	0 hpi	6 hpi	24 hpi	30 hpi	48 hpi
**ChibaC1T**	E2	7.625	100 (8/8)	100 (8/8)	25 (2/8)	0 (0/8)	0 (0/8)
**FukuokaT1**	E3	7.170	100 (8/8)	100 (8/8)	12.5 (1/8)	12.5 (1/8)	0 (0/8)
**Toyama16T**	E5	7.625	100 (8/8)	100 (8/8)	12.5 (1/8)	12.5 (1/8)	0 (0/8)
**Tokushima4T**	E7	8.375	100 (8/8)	100 (8/8)	0 (0/8)	0 (0/8)	0 (0/8)

[a] hpi, hours post-inoculation; numbers in parentheses, number of surviving chickens/total number of chickens

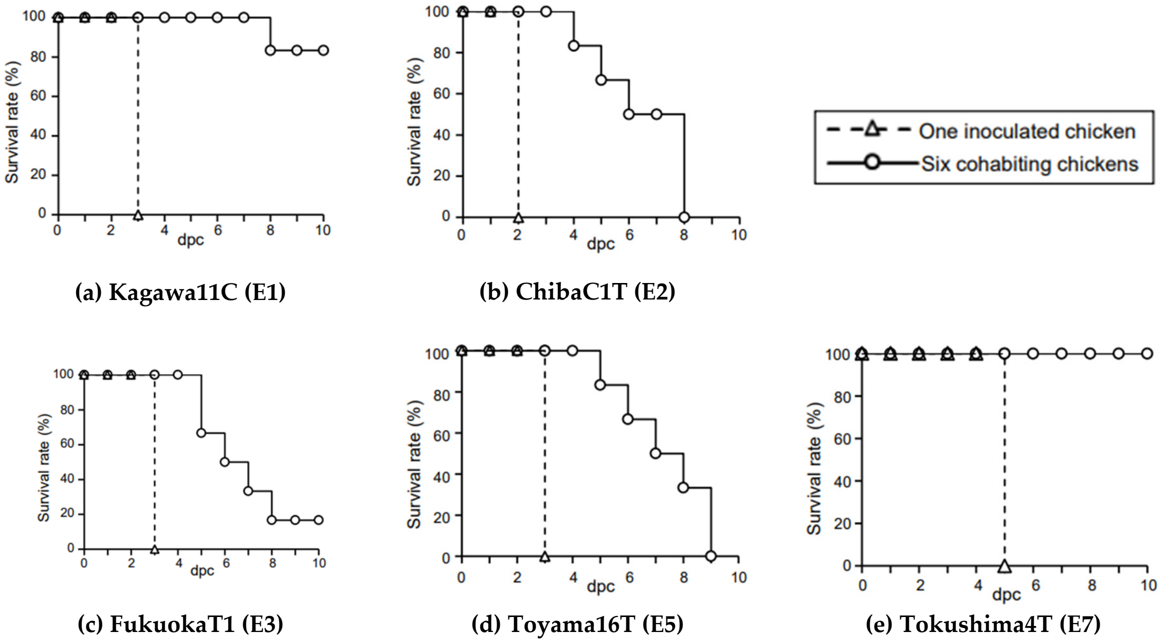

(a) Kagawa11C (E1) **(b) ChibaC1T (E2)**

(c) FukuokaT1 (E3) **(d) Toyama16T (E5)** **(e) Tokushima4T (E7)**

Figure S1. Survival rates of the chickens in the transmission experiments of (a) Kagawa11C, (b) ChibaC1T, (c) FukuokaT1, (d) Toyama16T, and (e) Tokushima4T until 10 days post-cohabitation (dpc). No chickens died after 10 dpc. Triangles represent virus-inoculated chickens, and circles represent cohabiting chickens.

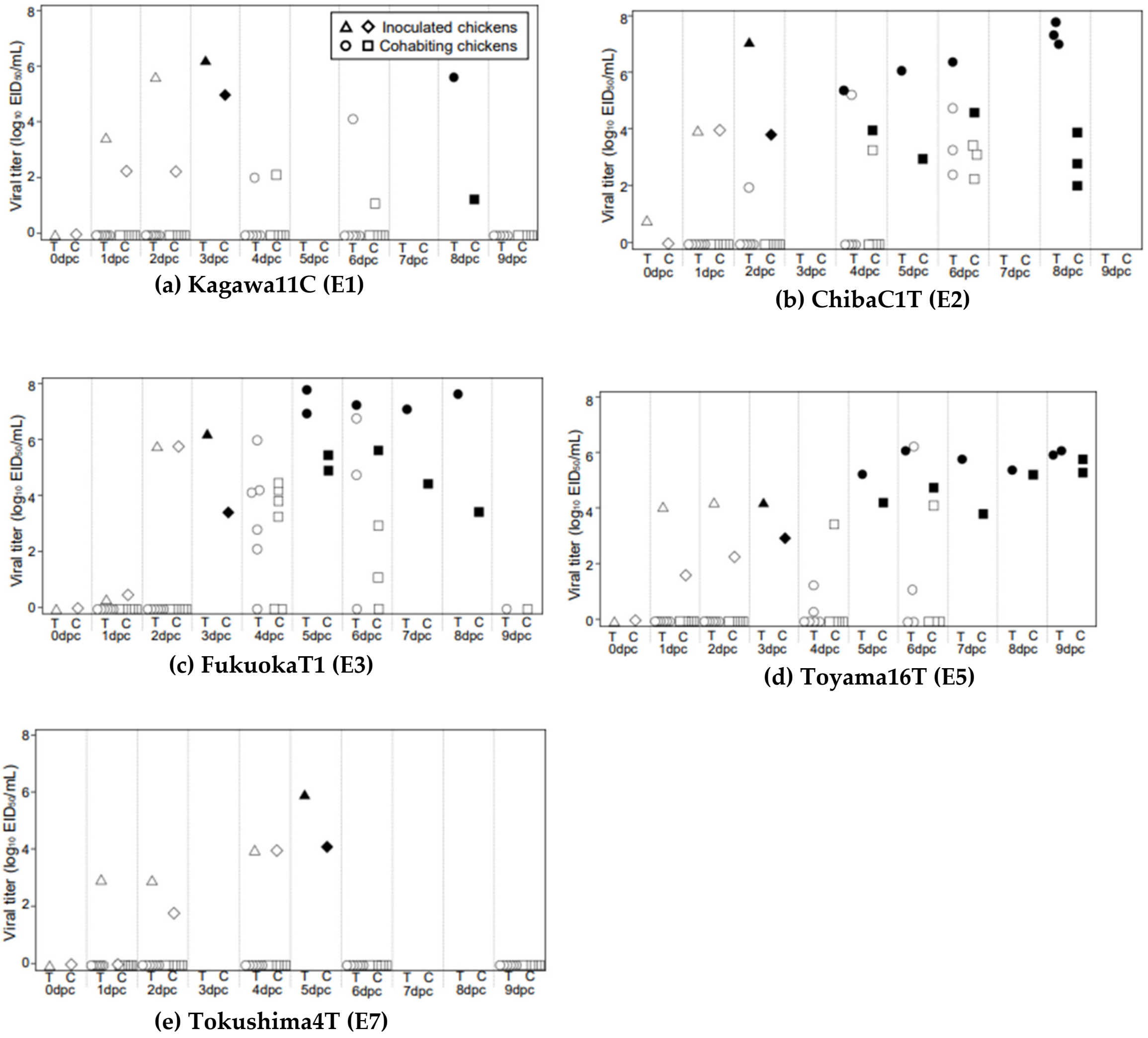

Figure S2. Viral titers in tracheal (T) or cloacal (C) swabs collected from chickens in the transmission experiments of (a) Kagawa11C, (b) ChibaC1T, (c) FukuokaT1, (d) Toyama16T, and (e) Tokushima4T until 9 days post-cohabitation (dpc). No viruses were detected in the tracheal and cloacal swabs collected from surviving chickens on 13, 16, and 20 dpc. Triangles and diamonds represent the viral titers of virus-inoculated chickens in tracheal and cloacal swabs, respectively. Circles and squares represent those of cohabiting chickens in tracheal and cloacal swabs, respectively. The color of symbols represents the conditions of chickens: white, live chickens; black, dead chickens.

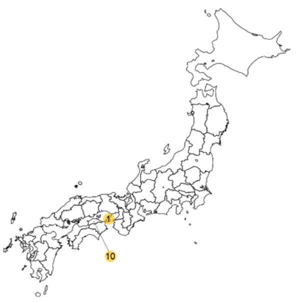

(a) E1 genotype

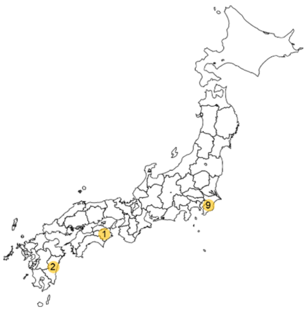

(b) E2 genotype

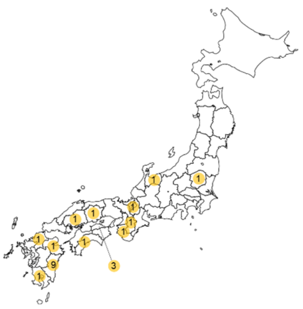

(c) E3 genotype

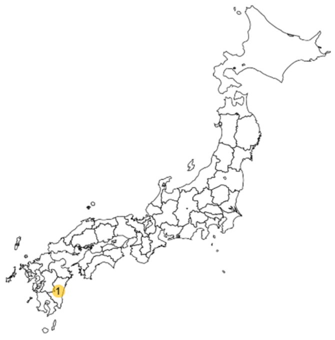

(d) E5 genotype

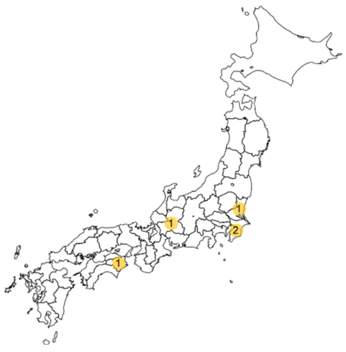

(e) E7 genotype

Figure S3. Locations and numbers of HPAI outbreaks in poultry farms in Japan during the winter of 2020–2021. The HPAI cases caused by viruses of the E1 (a), E2 (b), E3 (c), E5 (d), or E7 (e) genotypes, which include all 52 cases in poultry farms [13], are shown.
